# Aptamer Sensors for the Detection of Antibiotic Residues—A Mini-Review

**DOI:** 10.3390/toxics11060513

**Published:** 2023-06-07

**Authors:** Gang Liang, Le Song, Yufei Gao, Kailong Wu, Rui Guo, Ruichun Chen, Jianhui Zhen, Ligang Pan

**Affiliations:** 1 Institute of Quality Standard and Testing Technology, BAAFS (Beijing Academy of Agriculture and Forestry Sciences), Beijing 100097, China; lghbsd2006@163.com (G.L.);; 2 College of Environmental Science and Engineering, Hebei University of Science and Technology, Shijiazhuang 050024, China; 3 Ulanqab Agricultural and Livestock Product Quality Safety Center, Ulanqab 012406, China; wukailong_jn@163.com; 4 Datong Comprehensive Inspection and Testing Center, Datong 037000, China; guorui5617@126.com; 5 Shijiazhuang Customs Technology Center, Shijiazhuang 050051, China

**Keywords:** food safety, antibiotics, aptamer, biosensor, electrochemistry, colorimetry, fluorescence

## Abstract

Food security is a global issue, since it is closely related to human health. Antibiotics play a significant role in animal husbandry owing to their desirable broad-spectrum antibacterial activity. However, irrational use of antibiotics has caused serious environmental pollution and food safety problems; thus, the on-site detection of antibiotics is in high demand in environmental analysis and food safety assessment. Aptamer-based sensors are simple to use, accurate, inexpensive, selective, and are suitable for detecting antibiotics for environmental and food safety analysis. This review summarizes the recent advances in aptamer-based electrochemical, fluorescent, and colorimetric sensors for antibiotics detection. The review focuses on the detection principles of different aptamer sensors and recent achievements in developing electrochemical, fluorescent, and colorimetric aptamer sensors. The advantages and disadvantages of different sensors, current challenges, and future trends of aptamer-based sensors are also discussed.

## 1. Introduction

Food safety is critical to human health. With the improvement in living standards, food safety issues are receiving increased attention [1]. Food safety issues include pathogenic contamination, pesticides and veterinary drug residues, heavy metals and fungal toxin contamination, and illegal and adulterated use of food additives. It is a complex problem that contamination of food can occur at any stage between cultivation and consumption, involving the entire process from cultivation and breeding to production, processing, transportation, storage, and consumption. Therefore, governments worldwide have strengthened food safety monitoring and control of the entire chain from the “farm to the table” and have actively explored more effective ways of food safety supervision and management.

Antibiotics are a class of natural (produced and secreted from higher animals, plants, or microorganisms) or synthetic compounds with antimicrobial activity. They have been widely used in human and veterinary medicine and animal husbandry [2,3]. They are the main food pollutants detected in animal products such as meat, eggs, and milk. Studies have shown that the overuse and misuse of antibiotics in animal husbandry are serious, especially among enterprises or individuals engaged in animal husbandry. Driven by economic interests, they use functional drugs (such as antibiotics, sedatives, and growth-promoting drugs) in excessive amounts or in violation of regulations—to improve disease prevention and treatment effectiveness and to shorten the growth cycle. Long-term low-dose use of antibiotics can increase bacterial resistance, leading to the emergence of “superbugs” [4]. Antibiotics can pollute water sources and soil when released into the environment, posing significant ecological risks. Antibiotic residues can enter the human body via the food chain (meat, eggs, and milk) and harm human health [3,5,6]. Many countries and international organizations have established relevant regulations and standards regarding the use and maximum residue limits (MRL) of antibiotics. For example, the European Union has set the MRL of tetracyclines in milk at 100 μg/L, β-lactam antibiotics at 125 μg/L, macrolide antibiotics at 200 μg/L, and aminoglycoside antibiotics at 1500 μg/L [7]. China has also set 2191 residue limits for 267 veterinary drugs in animal and aquatic products, as well as in honey products [8], among which the MRL of tetracyclines is 1200 μg/kg, β-lactam antibiotics is 300 μg/kg, macrolide antibiotics is 2400 μg/kg, aminoglycoside antibiotics is 9000 μg/L, and quinolone antibiotics is 3000 μg/kg.

Antibiotic detection methods can be classified into physicochemical, microbial, immunological (i.e., ELISA), and biosensors [9,10,11]. At present, physicochemical methods, such as high-performance liquid chromatography (HPLC), gas chromatography (GC), liquid chromatography–mass spectrometry (LC-MS), and gas chromatography–mass spectrometry (GC-MS), are the most commonly used for antibiotic detection [12,13,14,15]. These physicochemical methods possess high stability, good reproducibility, high reliability, and high throughput detection ability. They have significantly improved the ability to analyze, detect, alert, and trace the risks of antibiotics in food. Nevertheless, these chromatographic methods suffer some inherent disadvantages, such as expensive instruments, complicated and time-consuming pretreatment, and the requirement of technical expertise, which limit their applications in rapid on-site antibiotics analysis. The demand for antibiotics residue detection in food production and processing is increasing to control the safety issues of animal-derived food and to uphold the bottom line of food safety. Thus, it is urgently required to develop simple, rapid, specific, and highly sensitive antibiotic-detection techniques.

With the developments in biosensor technology, aptamer sensors have evolved to detect pollutants such as pesticides, heavy metals, antibiotics, pathogenic bacteria, toxins, and POPs due to their high sensitivity, selectivity, and stability [16,17,18,19]. Since aptamers demonstrate a high affinity and specificity for their targets, aptamer biosensors have been widely used for antibiotic detection [20,21,22,23,24,25]. Especially with the insights into the functional aptamer structures and developments in the field of material science, elements such as split-aptamer [26], nicking–enzyme/signal label [27,28], and nanomaterials (such as nanogold, carbon nanomaterials, quantum dots, nano-oxides, and nano-enzymes [3,21,29,30]) have been gradually introduced to construct the sensing systems with significantly improved detection performance. This review discusses different applications of aptamer sensors for antibiotic detection using electrochemical, fluorescent, and colorimetric methods. The sensing mechanisms, present status, and application of aptamer sensors are also presented. Finally, future perspectives that can act as references for developments in aptamer sensor engineering for the sensitive detection of antibiotics are discussed.

## 2. Antibiotic Aptamers

**Figure 1:**
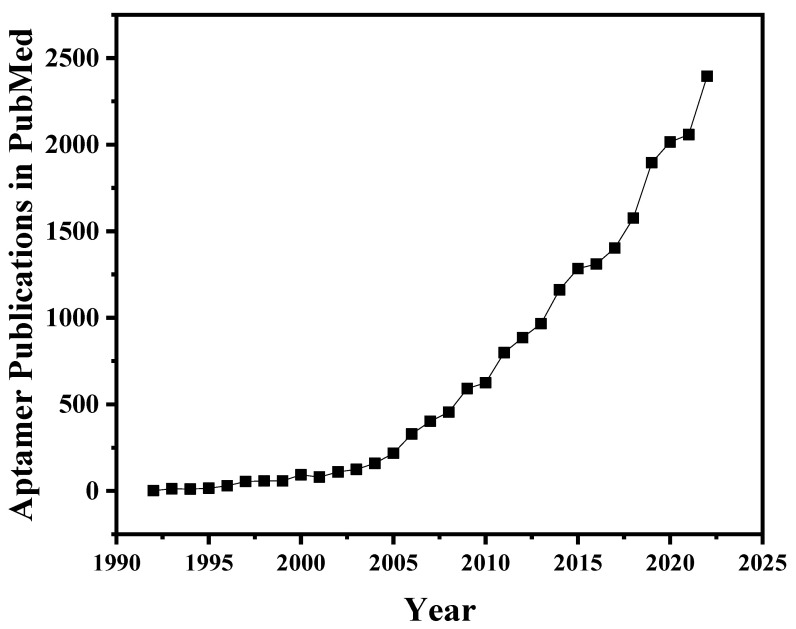
Annual publications in aptamer research (1992–2022).

Aptamers are typically defined as relatively short single-stranded deoxyribonucleic acid (DNA) or ribonucleic acid (RNA), which are usually obtained through a gold-standard methodology of systematic evolution of ligands by exponential enrichment (SELEX) technology. Typically, this technology includes an iterative selection procedure of binding, partitioning, recovery, and PCR amplification steps [26,31]. Like antibodies, aptamers bind specific targets. They have the advantages of quick chemical synthesis, versatile chemical modification, low production cost, relatively small physical size/molecular weight, good thermal stability, resistance to hydrolysis and inactivation, high specificity and affinity, broader targets than antibodies, and lack of immunogenicity [18]. Therefore, aptamers are also hailed as “artificial antibodies” [32]. After 30 years of research, hundreds of aptamers have been successfully screened and applied to analytical chemistry, biotechnology, biomedical and molecular biology [33]. Especially in the past decade, aptamers have seen unprecedented applications in biosensor research (Figure 1).

Aptamers can form secondary structures (hairpins, stem-loops, pseudoknots, bulge structures, I-motifs, and G-quadruplex) [34], which can fold into unique three-dimensional structures. The formations of the secondary structures play a critical role in recognition-based binding activity (affinity and selectivity); the unique three-dimensional structures provide sites capable of specific recognition and binding to their cognate targets [35]. Therefore, recent research has focused on constructing high-performance aptamer sensors by screening aptamers with high affinity and specificity that can distinguish between closely related molecules.

Research groups worldwide have contributed to maturing antibiotic aptamer screening technology, and remarkable progress has been made in sensing system construction [36]. For example, Choe et al. screened the enrofloxacin aptamer with Fe^3+^-IDA beads using traditional SELEX and capillary electrophoresis technology [37]. Lee et al. used biotin-labeled complementary oligonucleotides to capture the ssDNA library and linked them to agarose beads coated with streptomycin-binding protein. After incubation with danofloxacin, the danofloxacin-specific binding DNA sequence was released from the agarose bead, and consequently, the danofloxacin aptamer was screened [38]. Liu et al. established a novel aptamer screening method using magnetic separation technology with polydopamine magnetic nanobeads as separation carriers and lomefloxacin as a target molecule. The lomefloxacin aptamer showed high specificity to lomefloxacin by showing no affinity for the structurally similar molecules of enrofloxacin, ofloxacin, and norfloxacin [39]. Based on traditional SELEX technology, You et al. screened the ofloxacin DNA aptamer with functionalized graphene as the medium for binding and separation; the aptamer exhibited high affinity and specificity to ofloxacin [40].

Aptamers serve as recognition elements, which bind to specific antibiotics and exhibit excellent analysis performance. They have been widely utilized to construct sensing systems, such as interface state sensors (electrochemical, photoelectrochemical) and solution state sensors (colorimetric, fluorescent, chemiluminescent). With the rapid development of material science, the detection sensitivity of biosensing systems [41] was enhanced by introducing nanomaterials with unique chemical and physical properties (large surface area, high stability, superior electrical/optical performance, and good biocompatibility) [42]. These include carbon nanotubes, graphene, oxidized graphene, quantum dots, gold nanoparticles, and metal oxide nanoparticles, enhancing the detection performance.

Table 1 lists some of the antibiotic aptamers (sequences, base length, dissociation constants (*K*d)) published in the literature. Most aptamers exhibit high affinity to the target antibiotics with *K*d values in the nanomolar to micromolar range (see Table 1). Table 1 also shows that there may be more than two aptamers for one antibiotic. Conversely, the same aptamer might have more than one target antibiotic. Therefore, more effective aptamer screening methods are required to screen aptamers with stronger affinity and specificity for target antibiotics.

The applications of aptamer and nanomaterial/aptamer sensors in detecting antibiotics using electrochemistry, colorimetry, and fluorescence methods are discussed below.

## 3. Electrochemical-Based Antibiotic Sensors

### 3.1. Sensing Principle of the Electrochemical Sensor

**Figure 2:**
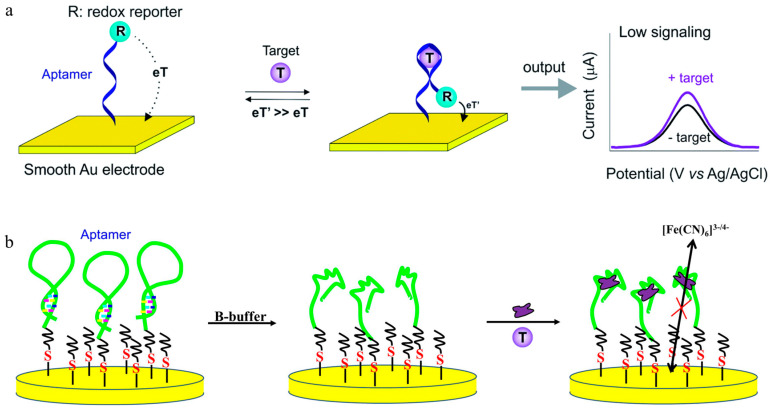
Scheme of the sensing principle for the Aptamer-based electrochemical sensor. (**a**) Structure switch of the aptamer leads to a short distance between the redox reporter and the gold electrode, resulting in an increased electrochemical current [43]. (**b**) The aptamer reacts with the target, forming a target/G-quadruplex complex and resulting in a charge-transfer resistance increase between the solution-based redox probe and the electrode surface [18]. (eT, electron transfer; B-buffer, binding buffer; EA, ethanolamine).

There are mainly two types of electrochemical detection techniques: voltammetry and impedance. Voltammetry measures the electrochemical current as the detection signal, and impedance measures the electrochemical impedance as the detection signal. The principle of electrochemical signal generation is that when the aptamer interacts with the target, there is a change in the distance between an electrically active molecule labeled with the aptamer (or complementary chain) and the electrode–electrolyte interface (or detachment from the interface), resulting in a variation in the electrochemical current (Figure 2a). Alternatively, the three-dimensional structure of the aptamer (or complementary chain) at the electrode interface changes, causing an increase/decrease in membrane electrochemical resistance (usually detected using potassium ferricyanide as a redox probe) (Figure 2b).

Electrochemical biosensing is rapid, sensitive, and portable. Compared with homogeneous sensing systems, such as colorimetry and fluorescence, the electrochemical aptamer film can eliminate non-target interference in the test solution via stepwise washing. However, the downsides of the electrochemical methods are that they involve complex procedures, such as activation of the electrode surface, material modification, and aptamer assembly. Thus, it is difficult for large-scale sample testing and analysis using these methods.

### 3.2. Application of the Electrochemical Aptamer Sensor for the Detection of Antibiotics

Generally, carbon electrodes (CE), glassy carbon electrodes (GCE), and metal electrodes (gold electrodes, GE) are the most widely used solid electrodes. CE and GCE usually require surface activation or modification before assembling the aptamers. Roushani’s group constructed an aptamer imprinting sensing film based on the GCE/graphene (GR)/silver nanoclusters and electrochemically detected chloramphenicol. The sensor measured the oxidation current signal of the chloramphenicol that was recognized and captured by the aptamer on the electrode surface; the sensor showed excellent detection ability with a sensitive detection limit of 3 × 10^−13^ mol L^−1^ [71]. Marty et al. constructed a disposable screen-printed aptamer sensor by activating the screen-printed carbon electrode with aminobenzoic acid by using a diazonium coupling reaction and by immobilizing the kanamycin aptamer on the surface by amidation reaction [72]. The prepared sensor realized rapid detection of kanamycin, with a detection limit of 2.3 × 10^−10^ mol L^−1^. Zhu modified GCE with a hierarchical porous flower-like Bi-BiOI@C composite (Bi-BiOI@C) and prepared a photoelectrochemical aptamer sensor for chloramphenicol; the sensor detected chloramphenicol with high sensitivity (detection limit of 7.9 × 10^−10^ mol/L) [73]. Qin et al. used silver sulfide/polydopamine/titanium dioxide, a nanotube-modified Ti sheet, as the electrode substrate to immobilize the thiolated aptamer onto the surface (through a S-Ag bond) to prepare a photoelectrochemical aptamer sensor for ofloxacin [47]. The aptasensor recorded a decrease in photocurrent due to the formation of the aptamer–ofloxacin complex with a detection limit of 7.5 × 10^−13^ mol L^−1^. Daprà et al. designed microfluidic double-channel sensor chips with the double-layer (tosylate doped poly (3,4-ethylenedioxythiophene) and the hydroxyl methyl derivative) film (conductive film and aptamer immobilized film) as two aptamers covalently immobilizing the substrate; the setup simultaneously detected penicillin G and kanamycin [74], with detection limits of 1 × 10^−10^ mol L^−1^ and 1 × 10^−8^ mol L^−1^, respectively.

Various other methods have been applied to improve the performance of sensors (sensitivity, stability, and specificity). For example, De waela et al. modified the GCE with gold nanoparticles and prepared a more stable aptamer-sensing film based on the Au-S bond, achieving high-sensitivity electrochemical detection of ofloxacin with a detection limit of 1 × 10^−9^ mol L^−1^ [75]. Taghdisi et al. assembled a methyl blue (MB)-labeled aptamer onto the electrode surface via a DNA hybridization interaction and then used it for enrofloxacin detection. After interacting with the enrofloxacin, the aptamer detached from the electrode interface, decreasing the MB electrochemical signal. Consequently, enrofloxacin could be detected with a detection limit of 1 × 10^−10^ mol L^−1^ [27]. Based on the enzymes-assisted dual-signal amplification strategy, Nie et al. constructed a tobramycin aptamer electrochemical sensor using a truncated aptamer with high affinity [76]. The sensor achieved specific detection of tobramycin with a detection limit of 5.13 × 10^−9^ mol L^−1^. Using target–aptamer binding to trigger multiple recycling amplifications, Wang et al. designed a signal-on aptamer sensor and achieved ultra-sensitive electrochemical detection of kanamycin with a detection limit of 1.3 × 10^−15^ mol L^−1^ [77]. González-Fernández et al., using magnetic separation technology, constructed a voltammetric detection of tobramycin using magnetic particles (MP) as a solid support for the inhibition assay [78]. Based on the competition between the MP-immobilized tobramycin and free tobramycin for the biotinylated aptamer, the quantification of tobramycin was achieved with a detection limit of 5 × 10^−6^ mol L^−1^. Xu et al. developed a novel sandwich-type electrochemical aptamer sensor by modifying graphene-polyaniline and polyamidoamine dendrimer-Au nanoparticle nanocomposites on the GCE surface (GO–PANI/PAMAM–Au/GCE), followed by loading of the kanamycin antibody (KAb), which was applied to capture kanamycin. With the biotinylated kanamycin binding aptamer (biotin-KBA) as the recognition element to KAb and kanamycin, a sandwich-type specific identification sensor for kanamycin was realized and further challenged for the detection of kanamycin by outputting a redox current from an electro-reduction reaction of H_2_O_2_ catalyzed by HRP, with a detection limit of 9.48 × 10^−6^ mol L^−1^ [79]. Combining the molecular imprinting technology’s specificity and aptamer’s target affinity, Lu et al. constructed a molecularly imprinted electrochemical aptasensor by co-depositing a zinc oxide and gold nanoparticle/reduced graphene oxide composite and electropolymerization of dopamine on an aptamer–amoxicillin complex film. Expectedly, the sensor showed excellent selectivity and sensitivity to amoxicillin, with an ultra-low detection limit of 3.3 × 10^−15^ mol L^−1^ [80].

With the development of materials science, functional materials have been used increasingly in the preparation of sensing membranes, significantly enhancing the interface conductivity and aptamer-loading capacity [81,82,83,84]. For example, Wang et al. synthesized a new covalent organic framework (COF) material (Py-M-COF) by polymerizing 1,3,6,8-tetra (4-formylphenyl) pyrene (Py) and melamine (M). They constructed an electrochemical aptamer sensor based on the Py-M-COF, detecting ampicillin with a detection limit of 1.2 × 10^−14^ mol L^−1^ [85]. Yan et al. constructed an electrochemical aptamer sensor based on high-capacity magnetic hollow porous nano-tracers coupling nuclease-assisted cascade target recycling methods; the sensor showed good performance for chloramphenicol and tetracycline with detection limits of 4.6 × 10^−10^ and 2.2 × 10^−10^ mol L^−1^ [86], respectively. Zhang et al. prepared a novel metal–organic framework (MOF) material ([Ru(bpy)_3_]^2+^@Ce-UiO-66/Mn:Bi_2_S_3_) by functionalizing the organic ligands and doping metal ions. An ofloxacin aptamer photoelectrochemical sensor was constructed using this method to detect ofloxacin [87], with a detection limit of 6 × 10^−12^ mol L^−1^. With Bi-doped ultrathin polymeric carbon nitride nanocomposites (Bi/CV-PCN) as the photoactive material, Xu et al. prepared a Bi/CV-PCN/indium tin oxide (ITO) photoelectrochemical electrode. A high-performance enrofloxacin aptamer sensor was then constructed [88]. Enrofloxacin was selectively detected based on the photocurrent signal change, with a detection limit of 9.18 × 10^−15^ mol L^−1^. Considering the high electron transfer capacity of the catalytic material, Song fabricated a TiO_2_/g-C_3_N_4_@AuNPs/GCE electrode and applied it to immobilize the aptamer via the Au-S bond between the thiol aptamer and AuNPs. The prepared aptamer sensor was applied for impedimetric amoxicillin detection, with a detection limit of 2 × 10^−10^ mol L^−1^ [89].

Table 2 lists the applications of electrochemical aptamer sensors for the detection of antibiotics, along with the strategy/recognition element, analytes and detection performance.

## 4. Colorimetric-Based Antibiotic Sensors

### 4.1. Sensing Principle of the Colorimetric Sensor

**Figure 3:**
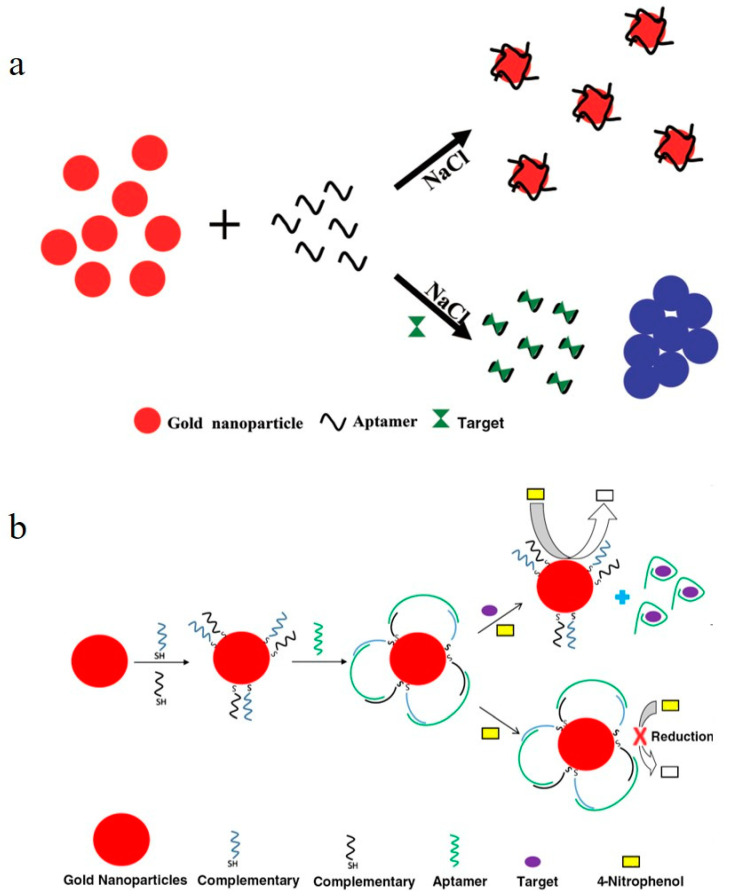
Sensing principle for the aptamer-based colorimetric sensor. (**a**) Target detection based on AuNPs aggregation induced by a high concentration of salt solution [90]. (**b**) Target detection based on the catalytic activity of AuNPs [101].

The colorimetric method is one of the most widely used sensing methods for aptamer biosensors because of its simplicity, speed, and visual detection. Gold nanoparticles (AuNPs), such as “AuNPs-aptamer-salts” and “AuNPs -aptamer-substrate”, are used as sensing probes in colorimetric systems due to their unique optical and catalytic properties. The sensing principles of the two colorimetric methods are the following: (1) aggregation of the aptamer modified/unmodified AuNPs, induced by a high concentration of a salt solution (resulting in a color change upon addition of salt) [90,91] (Figure 3a); (2) catalytic activity of the AuNPs on the colorimetric substrate in the presence of hydrogen peroxide (producing oxidation products with different UV–Vis absorption peaks) [92] (Figure 3b). Therefore, the target is detected because the aptamer–target interaction, which causes color changes in the AuNPs sensing system.

### 4.2. Application of Colorimetric Aptamer Sensors for Antibiotic Detection

Zhou et al. constructed an aptamer–AuNPs colorimetric system for specific, quantitative detection of ofloxacin by measuring the color change of the AuNPs system before and after the interaction of ofloxacin and the aptamer in a NaCl solution; the detection limit was 3.4 × 10^−9^ mol L^−1^ [93]. Wang et al. established a tetracycline detection method based on the tetracycline aptamer–AuNPs colorimetric system, with a detection limit of 2.693 × 10^−8^ mol L^−1^ [94]. Using cysteine-stabilized AuNPs as colorimetric probes, Luo et al. established a tetracycline aptamer–AuNPs colorimetric sensing system. After interacting with tetracycline, the AuNPs underwent a color change induced by a negative charge of the aptamer, leading to rapid detection of tetracycline with a detection limit of 8.78 × 10^−8^ mol L^−1^ [95]. Taking advantage of the reduction of nitrophenol by NaBH_4_ catalyzed by AuNPs, Lavaee et al. fabricated an aptamer sensor for ciprofloxacin. In the presence of ciprofloxacin, the aptamer detached from the AuNPs and triggered the catalytic activity of AuNPs, resulting in a color change from yellow to colorless [96]. The sensor showed good detection performance to ciprofloxacin with a detection limit of 1.2 × 10^−9^ mol L^−1^. Gold nanoclusters have catalytic activity and can catalyze oxidation of the substrate of 3,3′,5,5′-tetramethylbenzidine (TMB), resulting in a color change. Zhang et al. found that the catalytic activity of the gold nanoclusters was enhanced after interacting with tetracycline ligands [97]. A gold nanocluster–ligand–TMB probe colorimetric system was constructed, and a colorimetric method was established for tetracycline detection with a detection limit of 4.6 × 10^−8^ mol L^−1^.

To enhance the sensitivity of colorimetric detection, Liu et al. embedded the aptamer within a hairpin DNA structure, which would open in the presence of target molecules [28]. The partially released nucleic acids formed a double-stranded structure with nucleic acids labeled with magnetic beads (MB) and Pt probes at both ends. Based on this technique, a highly sensitive colorimetric detection method for kanamycin based on enzyme-cutting signal amplification was designed, with a detection limit of 4.1 × 10^−13^ mol L^−1^. Tian et al. utilized the repulsion effect of double-stranded DNA on gold nanoparticles to design a colorimetric aptamer biosensor based on aldehyde-functionalized magnetic beads and signal amplification by DNA hybridization chain reaction [98]. The change in the color system of the gold nanoparticles was used for the highly sensitive detection of kanamycin with a detection limit of 9 × 10^−10^ mol L^−1^.

The use of paper strips and magnetic bead systems as detection platforms has also been reported. Based on the split aptamer (SPA) strategy, they cut and obtained two aptamer fragments that have a selective binding affinity for enrofloxacin. By using the “sandwich” principle, they established an enrofloxacin amplification signal amplification strategy for paper strip and colorimetric detection methods [99,100], with detection limits of 2.8 × 10^−10^ and 6.95 × 10^−12^ mol/L, respectively. Wang et al. constructed a microplate colorimetric sensor based on the SPA strategy [101]. They coupled horseradish peroxidase (HRP) to the sensing membrane through the specific recognition of SPA1 and SPA2 to tilmicosin. The color change associated with the substrate catalysis was used to detect tilmicosin quantitatively, with a naked-eye detection limit of 1 × 10^−6^ mol/L. This method realized colorimetric analysis based on interfacial-state-sensing membranes, which improved the anti-interference ability of traditional colorimetric systems. In a separate study, Zhao et al. developed a colorimetric method for kanamycin and streptomycin detection based on the aggregation of the citrate-capped AuNPs induced by kanamycin and streptomycin instead of salt. They also reported that the adsorption of kanamycin and streptomycin by the AuNPs, instead of aptamer binding, was the reason for the color change [102].

The colorimetric method has the characteristics of simple, rapid, and detection with the naked eye, which is suitable for high-throughput screening and detection of pollutants in food and environmental samples. However, compared with the electrochemical detection technology, the colorimetric method currently uses homogeneous solution systems, where the sensing probes and targets are mixed in the same solution. Although it has good detection performance in buffer systems in the lab, it is susceptible to environmental interference in actual sample analysis, affecting the accuracy and reliability of the results. Thus, improving the anti-interference ability of the colorimetric method is an important area of research.

Table 3 lists the applications of colorimetric aptamer sensors for the detection of antibiotics, along with the strategy/recognition element, analytes and detection performance.

## 5. Fluorescent-Based Antibiotic Sensors

### 5.1. Sensing Principle of Fluorescent Sensors

**Figure 4:**
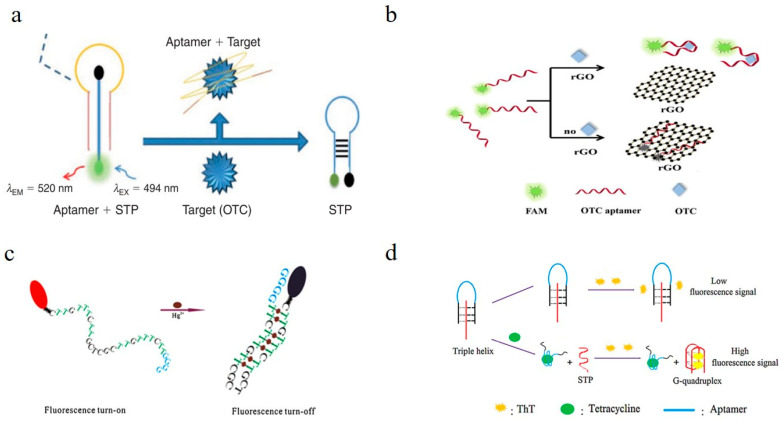
Sensing principle for the aptamer-based fluorescent sensor. (**a**) Target sensing based on aptamer molecular beacon labeled with fluorescence/quencher [103]. (**b**) Target sensing based on the fluorescence quenching by carbon nanomaterials [108]. (**c**) Fluorescence quenching of the aptamer by G-rich DNA chain [111]. (**d**) Interacting with the target, releasing G-quadruplex DNA that can bind with fluorescent molecules and that generates fluorescence signals [112]. (ThT, thioflavin T).

DNA/RNA aptamers do not have fluorescent properties. Thus, it is necessary to introduce fluorescent groups/molecules, fluorescent nanomaterials, and quantum dots to construct a fluorescence-based aptamer-sensing system. An aptamer fluorescent sensing system can be roughly divided into the following types: (1) Classical molecular beacon fluorescence sensing system by aptamer labeling of fluorescence/quencher groups [103,104,105] (Figure 4a): there are two added arm segments and the loop sequence of the signal transduction probe (STP), with an open configuration for the STP probe, where the fluorophore is far from the quencher and thus can give rise to a fluorescence event, and after binding of OTC to the aptamer, the STP is released, forming a stem loop structure that quenches the fluorescence. (2) Fluorescence-quenched sensing system of aptamers (or complementary chains) labeled with fluorescent groups and carbon nanomaterials (graphene/carbon nanotubes) [106,107,108] (Figure 4b): the FAM-modified aptamer can be adsorbed onto the rGO surface via π-π interactions, which induce them into fluorescence resonance energy transfer (FRET) proximity, leading to fluorescence quenching by rGO, while the formation of an OTC–aptamer complex decreases the exposure of nucleobases, which makes the dyes detach from the rGO surface, thus producing a restoration of fluorescence. (3) Fluorescence-quenched sensing system of aptamers (or complementary chains) labeled with fluorescent groups with specific DNA structures acting as quenchers (such as rich G alkaline base) [109,110,111] (Figure 4c): guanine has good electron-donating properties and can be used as a fluorophore quencher. As seen, the single signal probe shows a flexible random coil in aqueous solution, and the fluorophore and quencher are separated from each other, exhibiting strong fluorescence. When forming a duplex-like structure induced by interaction with the target, the structure holds the fluorophore and the guanine segment in close proximity to each other and leads to a remarkable decrease in fluorescence. (4) Fluorescence-sensing system of the specific structure DNA (such as G-quadruplex) binds with targets, generating fluorescence signals [112] (Figure 4d). By rationally designing the structure of the aptamers (such as single-stranded, double-stranded, hairpin, triplex, G-quadruplex structure, etc.) and the binding between the aptamer and the target molecule, antibiotics can be analyzed quantitatively by detecting the fluorescence signal change before and after an interaction.

### 5.2. Applications of Fluorescent Aptamer Sensors in the Detection of Antibiotics

Taghdisi et al. constructed an aptamer/aptamer complementary chain (ds-DNA)-SYBR gold–exonuclease III fluorescence sensing system. SYBR gold dye interacts with single-stranded DNA, producing strong fluorescent signals, while exonuclease III gradually hydrolyzes double-stranded DNA in the 3′→5′ direction. When streptomycin interacts with the aptamer, the ds-DNA structure unwinds into two single strands, limiting ds-DNA hydrolysis by exonuclease III, and displaying a strong fluorescent signal. As the streptomycin concentration increases in the solution, the fluorescent signal in the solution increases, resulting in streptomycin detection with a detection limit of 5.45 × 10^−9^ mol/L [113]. Ma et al. designed a ds-DNA fluorescent probe consisting of an aptamer and fluorophore group (FAM) labeled DNA1 and quencher group (BHQ1) labeled DNA2. Initially, DNA1 and DNA2 form a ds-DNA structure with the chloramphenicol aptamer, BHQ1 and FAM are “head-to-head” adjacent, and the FAM fluorescence in the system is in a quenched state. After chloramphenicol interacts with the aptamer, DNA1 and DNA2 are released into the solution, and the fluorescence recovers, resulting in chloramphenicol detection, with a detection limit of 2.16 × 10^−9^ mol/L [114]. Similarly, Jalalian et al. designed a triple-DNA structure with FAM and BHQ1 labeled at both ends of DNA1, which can interact with the hairpin DNA (aptamer) to form a triple DNA structure [115]. When the tetracycline interacts with the aptamer, the hairpin structure opens and releases DNA1. The free DNA1 forms a hairpin structure in the solution, and BHQ1 quenches the FAM. The system’s fluorescence signal decreases, and tetracycline can be detected with a detection limit of 2.09 × 10^−9^ mol/L. A fluorescence detection method for oxytetracycline was also constructed, with a detection limit of 1.67 × 10^−9^ mol/L [103].

Inspired by the above results, Chen et al. replaced DNA1 with a trimer structure labeled with the FAM and BHQ1 groups, with DNA2 containing a G-quadruplex structure [112]. After tetracycline interacts with hairpin DNA (aptamer), DNA2 is released into the solution, forming a G-quadruplex structure, which can interact with thioflavin T to produce strong fluorescence. Based on the fluorescence changes, tetracycline was detected, with a detection limit of 9.7 × 10^−10^ mol/L. Circular dichroism (CD) measurements have revealed that tetracycline aptamer can form a G-quadruplex structure in solution, which can specifically interact with fluorescent SYBR I to produce strong fluorescence signals. When tetracycline interacts with the aptamer, the G-quadruplex structure is destroyed, decreasing the fluorescence signal. Based on this principle, Yang et al. constructed an aptamer-SYBR I fluorescent sensing probe to detect tetracycline [116], with a detection limit of 2.25 × 10^−7^ mol/L. Ma et al. found that the kanamycin aptamer can change from a single-stranded structure to a G-quadruplex structure after interacting with kanamycin [117], confirming that thioflavin T can bind to the G-quadruplex to produce an enhanced fluorescence signal. Based on this, a kanamycin fluorescent sensing method was established, with a detection limit of 3 × 10^−10^ mol/L. Carbon nanomaterials, such as graphene and carbon nanotubes, absorb organic fluorescent molecules on their surface through π-π interactions, quenching their fluorescence. Based on this principle, Dolati et al. synthesized fluorescently labeled aptamer probes after screening the enrofloxacin aptamer [65] and constructed a graphene fluorescent-labeled aptamer sensing method. Based on the quenching of the fluorescent molecular signal by graphene, specific detection of enrofloxacin was achieved, with a detection limit of 3.7 × 10^−9^ mol/L.

C-rich DNA sequences can reduce the AgNO_3_ to silver nanoclusters, generating strong fluorescence at 620 nm (λem). Based on this, Hosseini et al. modified the tetracycline aptamer by extending a C-rich nucleic acid (C12) at one end of the DNA chain [118]. When AgNO_3_ was present in the solution, the C12 catalyzed the reduction of AgNO_3_ to generate fluorescent silver nanoclusters, resulting in a strong fluorescent signal (620 nm) in the system. The aptamer DNA and tetracycline formed a specific complex, which quenched the fluorescence of the silver nanocluster. Based on the fluorescent signal change, tetracycline was quantitatively analyzed with a detection limit of 1 × 10^−10^ mol/L. Using the aptamer–nanogold–SYBR green I fluorescence probe (fluorescence can be generated after SYBR green I interacts with the aptamer) [119], Yi et al. constructed a highly sensitive fluorescent method to detect ofloxacin—based on the competition between the aptamer, nanogold, and ofloxacin—with a detection limit of 3.4 × 10^−10^ mol/L.

Magnetic separation technology has been increasingly used to construct fluorescence systems to improve the sensitivity of fluorescence sensors. For example, Belal’s group cleaved a kanamycin aptamer into two fragments that could bind kanamycin with specificity. The two DNA fragments combined with ferromagnetic nanoparticles and CuS nanoparticles. After the recognition and capture of kanamycin by the two aptamer fragments, kanamycin was separated by magnetic separation technology. Using CuS to catalyze the fluorescent substrate after separation, a highly sensitive fluorescence method for kanamycin was established, with a detection limit of 2.6 × 10^−11^ mol/L [120]. Similarly, Liu et al. constructed a fluorescent-sensing system using aptamer-modified magnetic beads, and their complementary chains were labeled with up-converting fluorescent materials [121]. After specific interaction between the aptamer and its target, followed by magnetic separation, enrofloxacin was detected with a detection limit of 1.7 × 10^−10^ mol/L.

The fluorescence method has the advantages of rapid response, high sensitivity, flexible/versatile labeling, and multi-target detection. However, like the colorimetric method, the fluorescence method carries out detection in a homogeneous solution system. Thus, the fluorescence sensing probes and the tested targets are in the same solution, and the detection reliability can be affected by environmental factors such as heavy metals, pH, ion strength, and other interfering substances when analyzing actual samples. Like electrochemical methods, interface-state fluorescent-sensing membranes can be developed, improving the anti-interference ability and analysis accuracy of fluorescent detection; this is also the future development trend of the adaptive body fluorescence method. Combining molecularly imprinted polymer (MIP) with DNA aptamers (as a recognition technology) to achieve enhanced sensitivity and selectivity should also be investigated. Liu et al. have developed upconversion nanoparticles for detecting enrofloxacin based on molecularly imprinting and biotinylated enrofloxacin aptamers. With the combined characteristics of the specific molecular recognition properties of MIPs and aptamers, the recognition ability of these nanoparticles is further enhanced [122].

Table 4 lists the applications of fluorescent aptamer sensors for the detection of antibiotics, along with the strategy/recognition element, analytes and detection performance.

## 6. Conclusions and Prospects

Significant progress has been made in antibiotic aptamer screening, aptamer structure (second/tertiary) simulation and prediction, and construction (nanomaterial or) of aptamer sensor/sensing systems. However, there exist some limitations, such as, only a few antibiotic aptamers have been successfully screened and characterized. Although some of the aptamers exhibited very high target affinity, they also showed affinity to other antibiotics to a certain extent, producing non-specific signals when other similar compounds are present. Especially when the target concentration is low, non-specific signal interference cannot be ignored. Therefore, improving the specificity of aptamer screening technology or using neural network models for data processing/data fusion and then establishing target-specific recognition methods is also one of the future research directions for aptamer technology specificity.

Thus far, rapid and specific analysis of target antibiotics has been achieved using aptamer-sensing technology. However, existing research primarily focuses on detecting one antibiotic at a time and constructing single-channel sensing systems. The existing technology is also limited to detecting only a few antibiotics simultaneously. Thus, future research focusing on developing multi-channel sensing arrays is essential. Not only can they improve detection efficiency and reduce costs, but they can also enhance the fault-tolerant capability of sensing systems, increasing the credibility of the detection results.

Furthermore, although some of the aptamer sensors show a very low detection limit (DL), the DL was determined in buffer in the controlled lab environment, which is to say, all these references are only proof of concept, and none of these methods are directly applicable for self-monitoring or field control. Sometimes one real sample was analyzed, but it is not a full validation of the method in food products. Therefore, to verify the applicability of the sensor, a lot of work remains to apply these research biosensors to real sample analysis for antibiotic detection.

Although electrochemical technology has high sensitivity and strong anti-interference ability, it is limited by electrochemical instruments, making it difficult to prepare high-throughput aptamer array sensors. The colorimetry method has a simple, short preparation period and can achieve visual detection, but its accuracy and reliability might be affected by other substances in the solution. Considering the easier colorimetric signal acquisition, it is more suitable for developing array sensors. Solving the problem of interference resistance in the colorimetric system would promote aptamer-based detection products and should also become a future research direction. Aptamer-sensing technology has broad application prospects, which can be used for antibiotic screening, detection, and treatment. Future research should focus on improving the technology’s sensitivity, accuracy, and selectivity and should examine their applications in different fields.

## Figures and Tables

**Table 1:** Antibiotic-binding aptamers published in the literature.

Targets	Aptamer Sequences	Length (Bases)	Kd * (nM)	Refs.
Kanamycin A	GGGACTTGGTTTAGGTAATGAGTCCC	26	/	[43]
	TGGGGGTTGAGGCTAAGCCGA	21	78.8	[44]
Kanamycin B	TGGGGGTTGAGGCTAAGCCGA	21	84.5	[45]
Tetracycline	CGTACGGAATTCGCTAGCCCCCCGGCAGGCCACGGCTTGGGTTGGTCCCACTGCGCGTGGATCCGAGCTCCACGTG	76	70.7	[46]
	UUCACUGCUGCUUAAAGCCUAAAACAUACCAGAUCGCCACCCGCGCUUUAAUCUGGAGAGGUGAAGAAUUCGAC	74	1000	[47]
Oxytetracycline	GGAATTCGCTAGCACGTTGACGCTGGTGCCCGGTTGTGGTGCGAGTGTTGTGTGGATCCGAGCTCCACGTG	71	9.61	[48]
Tetracycline; Oxytetracycline; Doxycycline; Chlortetracycline	CGGTGGTG	8	1.067	[49]
Chlortetracycline	CGGTGGTGTTTTTTTTTTTTTTTT	24	/	[50]
	GGGAUCAUCACAGUGAAAAAAGAUCACACUGAAAAAAGAUCCC	43	2100	[51]
Tobramycin	TGGGGGTTGAGGCTAAGCCGA	21	103	[45]
	GATAATACGACTCACTATAGGAATGGATCCACATCTACGAACCTGTTATGTTTGAGAGAGGGTAGTCAATAGTGTTATGCCCGCTTCCCCCCTTTGGGGGTTCACTGCAGACTTGACGAAGCTT	124	150	[52]
	CGTCGACGGATCCATGGCACGTTACAGGTCGACG	34	56.8	[28]
Chloramphenicol	ACTTCAGTGAGTTGTCCCACGGTCGGCGAGTCGGTGGTAG	40	800	[53]
Streptomycin	TAGGGAATTCGTCGACGGATCCGGGGTCTGGTGTTCTGCTTTGTTCTGTCGGGTCGTCTGCAGGTCGACGCATGCGCCG	79	199.1	[54]
	CCCGTTTAAAGTAGTTGAGAGTATTCCGTTTCTTTGTGTC	40	6.07	[55]
	GCGCGCCCTAGGTACGATCGCGC	23	132.3	[56]
Penicillin G	CTGAATTGGATCTCTCTTCTTGAGCGATCTCCACA	35	/	[57]
	GGGAGGACGAAGCGGAACGAGATGTAGATGAGGCTCGATCCGAATGCGTGACGTCTATCGGAATACTCGTTTTTACGCCTCAGAAGACACGCCCGACA	98	/	[58]
	CACCAGTCAGACAGCACGGTGACTGGAGTGACGTCGGTACCTGAGATCGAGTGACGTCGGTACCTG	66	105.15	[59]
Ampicillin	CACGGCATGGTGGGCGTCGTG	21	9.4	[60]
	GCGGGCGGTTGTATAGCGG	20	13.4	[61]
	TTAGTTGGGGTTCAGTTGG	19	9.8	[60]
Benzylpenicillin	GGGTCTGAGGAGTGCGCGGTGCCAGTGAGT	30	384.3	[62]
amoxicillin	TTAGTTGGGGTTCAGTTGG	19	/	[63]
Danofloxacin	UCAGGCUCCUGUGAAGCAACCGAAUGGACUGA	32	1.81	[38]
Lomefloxacin	GACAGGCAGGACACCGTAACGGGTAGCCTCGCGACTCTTGAAGTTAATAGCCGGTGGTCCCTGGTACCTCCCTCCTCTTC	80	17.57	[39]
Ofloxacin	TGGCGCTTAGGTGTAATAACCTGAGGACGGCTTGG	35	130.1	[40]
Enrofloxacin	CCCATCAGGGGGCTAGGCTAACAGGTTCGGCTCTCTGAGCCCGGGTTATTTCAGGGGGA	59	188	[64]
	GCTGTGTGACTCCTGCAAGTCCGACATACCTTAGTGCCCTGATATAATGTAACACTATTGAGCAGCTGTATCTTGTCTCC	80	14.19	[65]
Ciprofloxacin	ATACCAGCTTATTCAATTCGATGGTAAGTGAGGTTCGTCCCTTTAATAAACTCGATTAGGATCTCGTGAGGTGTGCTCTACAATCGTAATCAGTTAG	97	/	[66]
Mapofloxacin; Pefloxacin; Ciprofloxacin; Ofloxacin; Levofloxacin; Deoxyfloxacin; Enrofloxacin; Moxifloxacin	ATACCAGCTTATTCAATTAGTTGTGTATTGAGGTTTGATCTAGGCATAGTCAACAGAGCACGATCGATCTGGCTTGTTCTACAATCGTAATCAGTTAG	99	0.11 **	[66]
Salfloxacin	CTCCGTGCGATCGCCGGGGACCGAAGAATCGTTCACATCG	40	48.08	[67]
Lincomycin	CGCGTGATGTGGTCGATGCGATACGGTGAGTCGCGCCACGGCTACACACGTCTCAGCGA	59	/	[68]
Azlocillin	CAGGAAGACAACTCCGACTAGAATTGATAATCAAGAATTCGTCTGGGGGGAATGTGCG	58	55	[69]
Neomycin	GGACUGGGCGAGAAGUUUAGUCC	23	115	[70]

* Dissociation constants; ** Ofloxacin.

**Table 2:** Applications of electrochemical aptamer sensors for the detection of antibiotics.

Strategy/Recognition Element	Analyte	Detection Limit		Ref.
		(nM)	(ug/L)	
Aptamer/silver sulfide/polydopamine/titanium dioxide nanotube/Ti sheet	ofloxacin	7.5 × 10^−4^	2.7 × 10^−4^	[47]
GCE/graphene(GR)/silver nanocluster/aptamer	chloramphenicol	3 × 10^−4^	9.7 × 10^−5^	[71]
SP carbon electrode/aminobenzoic acid	kanamycin	0.23	0.11	[72]
GCE/ Bi-BiOI@C/aptamer	chloramphenicol	0.79	0.255	[73]
Microfluidic double-channel sensor/tosylate doped poly(3,4-ethylenedioxythiophene) and the hydroxyl methyl derivative film	penicillin G	0.1	0.035	[74]
GCE/gold nanoparticles/aptamer	ofloxacin	1	0.36	[75]
Gold electrode/methyl blue (MB)-labeled aptamer	enrofloxacin	0.1	0.036	[27]
Enzyme-assisted dual-signal amplification	tobramycin	5.13	2.40	[76]
Target-aptamer binding triggered multiple recycling amplification	kanamycin	1.3 × 10^−6^	6.3×107	[77]
GCE/PAMAM–Au/GO–PANI/KAb	kanamycin	9.48 × 10^3^	4.60 × 10^−4^	[79]
Zinc oxide–gold nanoparticles/reduced graphene oxide composite /dopamine/aptamer–amoxicillin film	amoxicillin	3.3 × 10^−6^	1.2 × 10^−6^	[80]
Gold electrode/Py-M-COF/aptamer	ampicillin	1.2 × 10^−5^	4.2 × 10^−16^	[85]
Magnetic hollow porous nano-tracers coupling nucleases-assisted cascade target recycling methods	chloramphenicol	0.46	0.15	[86]
	tetracycline	0.22	0.098	
Aptamer/Ru(bpy)_3_]^2+^@Ce-UiO-66/Mn:Bi_2_S_3_	ofloxacin	6 × 10^−3^	2.17 × 10^−3^	[87]
Aptamer/Bi/CV-PCN/ITO	enrofloxacin	9.18 × 10^−6^	1.6 × 10^−6^	[88]
Aptamer/TiO_2_/g-C_3_N_4_@AuNPs/GCE	amoxicillin	0.2	0.073	[89]

**Table 3:** Applications of colorimetric aptamer sensors for the detection of antibiotics.

Strategy/Recognition Element	Analyte	Detection Limit		Ref.
		(nM)	(ug/L)	
Hairpin aptamer DNA/Enzyme-cutting signal amplification	kanamycin	0.00041	0.0002	[28]
Aptamer–AuNPs/NaCl	ofloxacin	3.4	1.23	[93]
Aptamer–AuNPs/NaCl	tetracycline	26.9	11.96	[94]
Aptamer/cysteine-stabilized AuNPs	tetracycline	87.8	39.02	[95]
Nitrophenol–NaBH_4_ /AuNPs	ciprofloxacin	1.2	0.40	[96]
gold nanocluster–ligand–TMB	tetracycline	46	20.44	[97]
Aldehyde-functionalized MB and DNA hybridization chain reaction signal amplification	kanamycin	0.9	0.44	[98]
Paper strips/split aptamer (SPA)	enrofloxacin	0.28	0.10	[99]
Magnetic bead/split aptamer (SPA)	enrofloxacin	0.00695	0.0025	[100]
Microplate plate/HRP–split aptamer (SPA)	tilmicosin	1000	869.2	[101]
Citrate-capped AuNPs/aptamer	kanamycin	90	43.6	[102]

**Table 4:** Applications of fluorescent aptamer sensors for the detection of antibiotics.

Strategy/Recognition Element	Analyte	Detection Limit		Ref.
		(nM)	(ug/L)	
Triple-helix molecular switch/hairpin aptamer/FAM-BHQ1	oxytetracycline	1067	491.28	[103]
Triple-helix molecular switch and G-quadruplex	tetracycline	0.97	0.43	[112]
Aptamer/aptamer complementary chain (ds-DNA)-SYBR Gold-exonuclease III	streptomycin	5.45	3.17	[113]
Aptamer/aptamer complementary chain (ds-DNA) labeled BHQ1 and FAM	chloramphenicol	2.16	0.70	[114]
Triple-helix molecular switch/FAM-BHQ1	tetracycline	2.09	0.93	[115]
G-quadruplex aptamer/SYBR I	tetracycline	225	100.0	[116]
G-quadruplex aptamer/ThT	kanamycin	0.3	0.15	[117]
Aptamer extending C-rich DNA sequence/silver nanoclusters	tetracycline	0.1	0.04	[118]
Aptamer–nanogold–SYBR Green I	ofloxacin	0.34	0.12	[119]
Aptamer/ferromagnetic nanoparticles/CuS nanoparticles	kanamycin	0.026	0.01	[120]
Aptamer-modified MB/up-converting fluorescent materials	enrofloxacin	0.17	0.06	[121]
Molecularly imprinting and biotinylated enrofloxacin aptamers	enrofloxacin	0.12	0.04	[122]
